# A Global Delphi Consensus on Acne and the Microbiome: Integrating International Expertise for Innovative Prevention and Therapeutic Strategies

**DOI:** 10.3390/ph19050697

**Published:** 2026-04-29

**Authors:** Marco Rocha, Leonel Fierro-Arias, Alison Layton, Vincenzo Bettoli, Ncoza Dlova, Eirini Merika, Thomas Dirschka, Pawinee Rerknimitr, Rakesh Newaj

**Affiliations:** 1 Dermatology Department, Universidade Federal de São Paulo, Sao Paulo 04023-062, Brazil; 2 Hospital General de México, Mexico City 06720, Mexico; 3 Dermatology Department, University of York, York YO10 5DD, UK; 4 Dermatology Department, University of Ferrara, 44121 Ferrara, Italy; 5 Dermatology Department, University of KwaZulu-Natal, Durban 4000, South Africa; 6 Chelsea and Westminster Hospital NHS Foundation Trust, London SW10 9NH, UK; 7 CentroDerm Clinic, Heinz-Fangman-Strasse 57, D-42287 Wuppertal, Germany; 8 Faculty of Health, University Witten-Herdecke, Alfred-Herrhausen-Strasse 50, D-58455 Witten, Germany; 9 Dermatology Department, Chulalongkorn University, Bangkok 10330, Thailand; 10 Arwyp Medical Centre, Johannesburg 1619, South Africa

**Keywords:** acne, microbiome, global consensus

## Abstract

Acne is a prevalent dermatological condition occurring globally and influenced by a variety of endogenous and exogenous factors. The microbiome and its contribution to skin disease have been increasingly explored, along with the influence of the exposome and host immune responses on this complex microbial system. Nine experts from different countries in Africa, America, Asia, and Europe gathered to harmonise definitions, identify key pathogenic and protective microbial strains, and prioritise the factors that most significantly impact the skin’s microbiome in the context of acne. Opportunity areas on the role of the microbiome in the prevention, treatment, recurrence, and sequelae avoidance in acne were identified. The relationships between current treatments and the diversity of the microbiome were described. Current microbiome-targeted strategies were assessed, including practical considerations of innovative future perspectives. The panel discussions emphasise the urgent need for universally adaptable guidelines encompassing alternatives to oral antibiotic therapies, in light of increasing antimicrobial resistance and the significant burden of treatment-related adverse events.

## 1. Introduction

Acne vulgaris remains one of the most common dermatological conditions worldwide, affecting individuals across diverse age groups and contributing significantly to psychosocial morbidities such as anxiety, depression, and even suicidal behaviour [1]. Although acne has been recognised since ancient times, the pathogenic role of bacteria was first suggested by Unna in 1896, with Sabouraud subsequently isolating and naming *Bacillus acnes*. Later, *Bacillus acnes* was renamed *Propionibacterium acnes* in the 1940s and, following further taxonomic refinement in 2016, was reclassified as *Cutibacterium acnes*; nevertheless, the term *Propionibacterium acnes* continues to be used and remains taxonomically valid [2,3]. In recent years, advances in metagenomics and immunology have illuminated the complex interplay between the skin microbiome and host physiology. The exposome and the immune response are now recognised as key factors influencing this microbiome, which is unique to each individual and varies according to body site [4,5]. In this context, eubiosis refers to a balanced mutualism between the host and its microbiota. However, dysbiosis, disrupting this homeostasis, may trigger or exacerbate inflammatory skin diseases [6]. Recent research has expanded the traditional understanding of acne pathogenesis by demonstrating that alterations in the lipid environment, disruption of skin commensals, and changes in the distribution of *C. acnes* strains contribute to epidermal barrier impairment, characterizing the dysbiosis observed in the disease [7,8]. A systematic review has underscored the intricate role of microbes in acne and highlighted the necessity for personalised, microbiome-based strategies [9]. The imbalance between beneficial and harmful bacterial strains appears to be more critical in acne pathogenesis than the mere overgrowth of specific microbes [10], and it is increasingly recognised that conventional treatments may either support or hinder the restoration of a healthy microbial composition [11].

### 1.1. Delphi Consensus Process and Methodological Approach

A modified Delphi methodology [12] was employed to achieve structured expert consensus on the role of the skin microbiome in acne pathogenesis, prevention, and treatment. This approach was chosen to systematically integrate current scientific evidence with expert clinical judgment in an area where high-level evidence remains limited. The literature search strategy included PubMed and Embase databases using combinations of the following keywords: “acne”, “skin microbiome”, “*Cutibacterium acnes*”, “exposome”, “skin barrier”, “probiotics”, “prebiotics”, “postbiotics”, and “gut–skin axis”. Studies published in English within the last 10–15 years were prioritized. Both clinical and mechanistic studies were considered. Exclusion criteria included non-peer-reviewed sources, studies lacking methodological clarity, and publications not directly related to acne or microbiome interactions.

An international panel of nine dermatologists with recognized expertise in acne and microbiome research was convened, representing diverse geographic regions (Brazil, Germany, Italy, Mexico, South Africa, Thailand, and the United Kingdom). The process was conducted between May and September 2024 and consisted of iterative rounds of evidence review, structured surveys, and group discussions.

Initially, a targeted narrative literature review was performed using PubMed and Embase databases to identify relevant studies on acne, the skin microbiome, the exposome, and microbiome-targeted interventions. Based on this review, a preliminary set of statements was developed by the coordinating author and refined collaboratively by the panel.

The Delphi process consisted of two formal electronic survey rounds, complemented by live discussions (virtual and in-person). In each survey round:

- Panelists independently rated their level of agreement with each statement using a structured scale.
- Responses were collected anonymously to minimize the influence of dominant opinions and reduce group bias.
- Statements not reaching consensus were revised based on aggregated feedback and redistributed in subsequent rounds.

Consensus was predefined as ≥75% agreement, consistent with commonly accepted thresholds in Delphi-based research. Statements reaching this threshold were retained, while those below the threshold were either reformulated or excluded after discussion.

The first round focused on foundational concepts (e.g., microbiome contribution to acne pathogenesis, influence of skincare and exposome), while subsequent rounds addressed emerging and more controversial topics, such as microbiome-targeted prevention strategies, pre/pro/postbiotic interventions, and the gut–skin axis.

Importantly, the panel explicitly differentiated between:

- Statements supported by robust clinical or mechanistic evidence;
- Statements reflecting expert consensus in areas of limited or evolving evidence.

**Figure 1:**
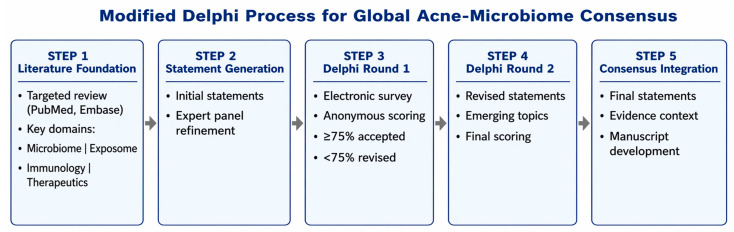
Modified Delphi process used to develop the global consensus on acne and the microbiome. A structured, multi-step approach was employed, beginning with a targeted narrative (non-systematic) literature review intended to support statement development rather than provide a comprehensive systematic synthesis. Initial statements were generated and refined by an international panel of experts. Two rounds of anonymous electronic surveys were conducted, interspersed with virtual and in-person discussions. Consensus was predefined as ≥75% agreement. Statements not reaching consensus were revised and re-evaluated in subsequent rounds. The final set of statements represents a synthesis of current evidence and expert interpretation, highlighting both well-established concepts and emerging areas of research. This structured approach enhances methodological transparency and aligns with established recommendations for Delphi-based consensus research. It is important to emphasize that consensus reflects expert agreement and should not be interpreted as a surrogate for high-level evidence. Statements related to emerging concepts, such as microbiome-targeted prevention strategies, should be considered hypothesis-generating and require further validation.

The wording of the consensus statements was preserved as generated during the Delphi process and was not modified after consensus was reached. Therefore, agreement levels reflect expert opinion and should not be interpreted as a direct measure of the strength of evidence. A summary of the process, including statement selection and progression across rounds, is presented in Figure 1.

### 1.2. Pathogenesis: The Role of the Microbiome in Acne

The skin microbiome is composed of a diverse community of resident microorganisms—including bacteria, fungi, viruses, and mites—that coexist in a delicate balance with the host. In healthy individuals, the core microbiome is represented by 19 phyla, with the majority of commensal bacteria belonging to *Actinobacteria* (51.8%), *Firmicutes* (24.4%), *Proteobacteria* (16.5%), and *Bacteroidetes* (6.3%) [13]. In addition to bacterial populations, the skin microbiome also includes fungi (e.g., *Malassezia* spp.), viruses (including bacteriophages), and mites, all of which may contribute to the dynamic ecosystem of the skin. Although their role in acne is less well characterized, emerging evidence suggests that inter-kingdom interactions may influence inflammation, microbial competition, and barrier function. The distribution of these microorganisms varies according to skin region and microenvironment; for instance, *Cutibacterium* spp. thrive in lipid-rich, anaerobic conditions, while drier regions exhibit a mixture of phyla and moist areas favour the proliferation of *Staphylococcus* spp. and *Corynebacterium* spp. [14] Numerous studies have shown that both increases and decreases in the abundance of specific microbial communities can negatively impact skin health and facilitate the development of various dermatological conditions [15]. Traditional culture-based methods have underestimated this diversity; however, advances in amplicon and shotgun metagenomic sequencing now enable a more precise characterisation of these communities and their functional roles [16,17].

In the context of acne, quantitative differences in *C. acnes* between affected and healthy skin are not consistently observed; qualitative differences—specifically, variations in the strain composition and genetic characteristics—are critical. Certain strains of *C. acnes* contribute to skin health by producing short-chain fatty acids and bacteriocins that inhibit pathogens, whereas others, such as *C. acnes* type IA1, are associated with heightened inflammation and increased biofilm formation, and can induce the release of proinflammatory cytokines such as IL-1β, IL-6, IL-8, TNF-α, IL-12, and IL-23, contributing to both innate and adaptive immune responses in acne [2,18,19]. Importantly, these findings should be interpreted within the broader context of microbial community interactions, where *C. acnes* acts as a key—but not isolated—component of a complex ecological network. The use of antimicrobial agents may inadvertently promote more virulent strains (IA1 and IA2) and contribute to the emergence of antibiotic resistance through mutations in the 16S and 23S rRNA genes, as well as enhanced biofilm production [14,15,20,21]. Conversely, strains such as *C. acnes* type IB are not linked with acne, and type III has been found to possess the highest pro-inflammatory potential via upregulation of proteinase-activated receptor-2 (PAR-2), tumour necrosis factor-alpha (TNF-α), matrix metalloproteases (MMPs), and tissue inhibitors of metalloproteases (TIMPs) [19,22,23,24,25]. According to Dagnelie et al., patients with severe acne exhibit a predominance of IA1 strains and a reduction in overall strain diversity. This shift may trigger an innate immune response and inflammation [26]. In vitro studies further support that restoring microbial diversity can suppress inflammation by downregulating innate immune pathways. [27] Comparative genomic analysis of 82 *C. acnes* strains revealed lineage-specific genetic elements that may explain its dual role as a skin commensal and a pathogen, highlighting the importance of strain-level microbiome analysis for personalized acne therapies [28]. Moreover, cutaneous and gut microbes are now recognised as critical factors in maintaining skin health or exacerbating disease, opening avenues for novel therapeutic interventions [29].

### 1.3. Relationship Between the Microbiome and the Skin Barrier

**Figure 2:**
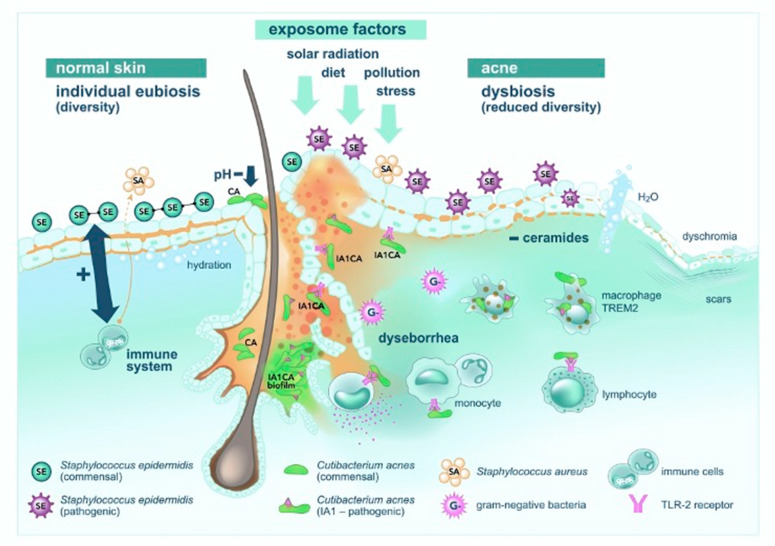
The progression from eubiosis to dysbiosis and the detrimental effects on acne development. From left to right: Commensal *Staphylococcus epidermidis* and *Cutibacterium acnes* strains promote immune synergy, pH homeostasis, balanced sebaceous activity, and an intact epidermal barrier. Progressing toward the right: cumulative exposomal and intrinsic factors disrupt this equilibrium, precipitating dysseborrhea alongside compromised barrier integrity, pathogenic colonisation, and biofilm formation. Pathogenic *Cutibacterium acnes* (IA1) further activates immune pathways, heightening intercellular signalling and fostering follicular disarray, microcomedone formation, and clinical lesion development. This pathological cascade predisposes tissue to subsequent scarring and dyschromia. This figure represents a conceptual model based on current evidence and expert interpretation rather than a direct representation of a single dataset.

Alterations in the skin barrier have been directly associated with dysbiosis, a common feature of inflammatory skin disorders. In acne, barrier dysfunction may contribute to the predominance of *C. acnes* phylotypes IA and II, which exhibit enhanced biofilm formation, whereas phylotypes IB and III are more typically associated with healthy skin [8,30,31]. Biofilms enable *C. acnes* to persist and confer resistance to antibiotic therapy, suggesting that anti-biofilm agents might play a role in restoring microbial balance. Furthermore, acne patients often exhibit intrinsic alterations in the skin barrier, such as increased filaggrin expression and reduced ceramide levels, which in turn affect transepidermal water loss and overall permeability [25]. Restorative interventions using moisturisers or cosmetics enriched with probiotics have been shown to improve barrier function, further supporting the potential of microbiome-targeted therapies in acne management [32,33,34,35] (Figure 2). From a clinical perspective, strategies aimed at restoring the skin barrier—such as the use of ceramide-containing moisturizers, non-irritating cleansers, and microbiome-supportive dermocosmetics—may help reduce inflammation, improve treatment tolerability, and potentially enhance therapeutic outcomes in acne patients.

### 1.4. The Microbiome and Acne Severity

Multiple factors influence acne severity, including the predominance of more virulent *C. acnes* phylogroups, altered diversity of *Staphylococcus epidermidis*, and shifts in overall microbial diversity. Increased alpha diversity and higher proportions of Gram-negative bacteria such as *Faecalibacterium*, *Klebsiella*, *Odoribacter*, and *Bacteroides* have also been associated with more severe forms of acne. These observations underscore the complexity of host–microbe interactions and highlight the need for further studies to elucidate the precise mechanisms by which microbial dysbiosis influences disease severity. Recent studies using metagenomic approaches have further demonstrated that decreased microbial diversity and dominance of specific virulent strains correlate with increased inflammatory lesion counts, supporting a quantitative relationship between dysbiosis and acne severity [36].

### 1.5. The Microbiome in Acne-Induced Hyperpigmentation (AIH) and Scarring

Although certain *C. acnes* strains with heightened inflammatory potential are known to contribute to lesion formation, the mechanistic links between microbial dysbiosis, sustained immune activation, and the development of acne-induced hyperpigmentation and scarring remain insufficiently elucidated, highlighting a critical gap in our understanding of how microbiome alterations influence long-term tissue remodeling [37,38]. The dyschromic consequence is particularly prevalent in individuals with skin of color, and current consensus guidelines recommend early intervention with treatments such as hydroquinone, azelaic acid, chemical peels, or antioxidants to mitigate its development [39,40]. More recently, isobutylamido-thiazolyl-resorcinol (Thiamidol), a potent inhibitor of human tyrosinase, has emerged as a promising agent for addressing acne-induced hyperpigmentation [41].

### 1.6. Impact of Conventional Acne Treatments on the Skin Microbiome

Traditional acne treatments—including topical benzoyl peroxide (BPO), systemic isotretinoin, and both oral and topical antibiotics—effectively reduce lesion counts, yet they can also impact the skin microbiome. BPO exerts a bactericidal effect that reduces both antibiotic-sensitive and resistant strains of *C. acnes* [42]. Oral isotretinoin reduces sebum production, leading to a secondary decrease in *C. acnes*, and has been associated with significant increases in both alpha- and beta-diversity of the skin microbiome, changes that correlate with clinical improvement [43]. However, the widespread use of antibiotics raises concerns regarding the promotion of antibiotic-resistant strains, gastrointestinal side effects, and potential associations with inflammatory bowel diseases [23,44,45]. In addition, antibiotics may alter non-cutaneous microbial populations and have even been hypothesised to trigger upper respiratory tract infections, although conclusive evidence is still lacking [45]. Notably, the persistence of virulent *C. acnes* strains may not depend solely on microbial resistance; lipid components of sebum, such as oxidized squalene—abundant in acne lesions—can modulate keratinocyte gene expression and innate immune responses, potentially perpetuating inflammation even in the presence of antimicrobial therapy [46,47].

### 1.7. Positive Interventions and Cosmetic Adjuncts

While it may seem counterintuitive to moisturise oily skin, the use of non-comedogenic moisturisers—especially those containing anti-inflammatory agents such as ceramides and niacinamide—is recommended as an adjunct to conventional acne treatments to prevent irritation and transepidermal water loss [33,48]. Although high-quality randomized controlled trials remain limited, emerging evidence and expert consensus suggest that these interventions may provide clinical benefits as adjunctive or maintenance strategies [34,38].

### 1.8. Lifestyle Practices, the Exposome, and the Gut–Skin Axis

External and internal factors, collectively called the exposome, influence the skin microbiome. They include sun radiation, air pollution, tobacco smoke, nutrition, and even cosmetic products [49,50]. Similarly, the gut microbiome, which is affected by ageing, diet, illness, antibiotic use, and travel, plays a significant role in overall health. A complex, bidirectional relationship exists between the gut and skin microbiomes, mediated by systemic immune and inflammatory pathways [29,51,52,53,54,55]. Dietary components and probiotics have been shown to influence the gut microbiome [56,57,58,59], which can affect the skin microbiome and potentially modulate acne [60]. Although the role of specific dietary factors in acne remains controversial—with dairy products, chocolate, and saturated fats often implicated—the evidence is conflicting due to diverse diet compositions and cultural differences [61]. Epidemiological studies support that high-glycaemic load diets and increased dairy protein consumption, common in Western diets, may exacerbate acne [61,62], although definitive validation of these associations is elusive. Additionally, cleansing practices and the use of moisturisers can alter the skin’s microbiome and biophysical properties; excessive scrubbing may disrupt the skin barrier and activate innate immunity, leading to acne aggravation [63,64]. Therefore, cleansers should be formulated to be non-comedogenic, non-acnegenic, non-irritating, and non-allergenic, with a preference for non-ionic, fragrance-free products that rinse easily [65]. Moisturisers designed for acne often contain anti-inflammatory ingredients. They may include active anti-acne agents like salicylic acid alongside components such as dimethicone or glycerine, and occasionally botanical extracts with anti-inflammatory properties [32].

### 1.9. Results of the Global Delphi Process

Overall, the Delphi process demonstrated a strong and consistent consensus regarding the central role of the microbiome in acne pathogenesis, as well as the significant influence of skincare practices and exposome-related factors on this complex ecosystem. Emerging concepts, including microbiome-targeted prevention and maintenance strategies, also achieved moderate to high levels of agreement, reflecting growing scientific and clinical interest despite the current limitations in high-level evidence.

This structured process culminated in a series of consensus statements, summarized in Table 1. In the first electronic survey, several key statements reached a high level of agreement (≥75%), including the recognition of the microbiome as a crucial contributor to acne pathogenesis (100%), the impact of skincare practices on microbiome composition (100%), and the direct influence of the exposome—encompassing lifestyle, climate, diet, and pollution—on the skin microbiome (100%).

Furthermore, the panel unanimously agreed that microbiome-targeted treatments should be adopted for acne management (100%). Moreover, other statements regarding the influence of the microbiome on acne severity (89%), the direct impact of the gut–skin axis (89%), the potential benefits of probiotics (89%), and the inclusion of prebiotics, probiotics, and postbiotics in acne therapy (89%) also reached high consensus. Newer strategies were similarly debated, such as microbiome-targeted approaches for primary prevention (78%) and maintenance to avoid acne relapses (75%). In a subsequent survey round, additional statements reached consensus: the microbiome’s role in acne-induced hyperpigmentation (100%), the positive influence of topical prebiotics in acne treatment (100%), the impact of the microbiome on acne-induced scarring (89%), and the influence of high-glycaemic dietary habits on the composition of the skin microbiome and acne pathogenesis [62].

### 1.10. Practical Considerations and Innovative Future Perspectives

Future research should prioritize the generation of high-quality evidence through well-designed randomized controlled trials evaluating microbiome-targeted interventions. In parallel, efforts should focus on the standardization of microbiome assessment methods, the identification of clinically relevant microbial signatures, and the evaluation of the long-term safety and efficacy of emerging therapies. Within this context, and considering the limitations and potential drawbacks of conventional acne treatments, the panel explored innovative microbiome-targeted strategies as both adjunctive approaches and potential avenues for primary prevention. The experts discussed the promise of interventions aimed at modulating the cutaneous microbial ecosystem—approaches that could selectively target pathogenic strains while preserving beneficial microorganisms [66,67]. In light of emerging evidence on the gut–brain–skin axis [51,53,55], strategies that include oral and topical prebiotics, probiotics, and postbiotics are being investigated to restore microbial balance and attenuate early inflammatory responses. Although the statement that “microbiome-targeted strategies should be adopted as primary prevention of acne” achieved strong consensus, the underlying mechanisms and clinical applicability of such strategies remain an area of active research and may represent a promising approach for primary prevention, although current evidence remains limited [68]. Detailed discussions centred on the potential of topical formulations containing prebiotics to enhance skin barrier function and suppress pathogenic biofilm formation despite the relatively limited supporting data (100% consensus).

Additional innovative approaches discussed include bacteriotherapy and phage therapy. Topical bacteriotherapy—using live beneficial bacteria to correct dysbiosis—has demonstrated feasibility in preliminary studies, while oral and topical pre-, pro-, and postbiotics may act through the gut–skin axis to offer systemic benefits [6,69,70,71]. As our understanding of the complex roles of different *C. acnes* strains evolves, further efforts in microbiome manipulation, including bacterial transplantation, are anticipated to expand therapeutic options [72,73]. Although early open-label studies using donor microbiome mixtures have shown potential benefits, robust randomised controlled trials are necessary to validate these findings [74,75].

Phage therapy represents another promising avenue by selectively targeting pathogenic *C. acnes* strains with lytic phages, thereby reducing inflammation and biofilm formation while preserving the commensal flora. Despite its advantages, challenges remain regarding phage delivery to follicular structures, the potential for resistance, and the inflammatory response elicited by lysed bacteria [69,71]. Vaccine development targeting specific virulence factors of *C. acnes*, such as the CAMP factor, has been pursued for decades, and recent advances—including an mRNA vaccine trial initiated in 2024—offer hope for long-term modulation of the host–microbiome interaction without disturbing microbial homeostasis [76,77,78]. However, these approaches remain experimental, and significant challenges persist, including variability in host response, delivery mechanisms, long-term safety, and regulatory considerations, which currently limit their translation into routine clinical practice.

## 2. Discussion

Given the involvement of industry-supported research in this field, interpretations of microbiome-targeted strategies should be approached with caution. The present consensus aims to balance current evidence with expert interpretation, acknowledging potential limitations and areas of uncertainty.

This global Delphi consensus brings together international clinical expertise and contemporary scientific insights to provide a comprehensive framework for understanding the multifaceted role of the skin microbiome in acne. The perspectives presented herein reflect expert agreement in areas where the evidence base remains heterogeneous or is still evolving, and should therefore be interpreted within the context of current scientific knowledge rather than as definitive evidence-based recommendations. By structuring the manuscript around an iterative Delphi process, the panel not only reaffirms well-established concepts but also highlights emerging and innovative strategies, particularly those related to microbiome modulation as a potential avenue for primary prevention.

Importantly, the role of the microbiome in acne cannot be fully understood in isolation, but rather within the broader context of the follicular immune microenvironment. The infundibulum serves as a critical interface between the hair follicle and the external environment, where resident Langerhans cells regulate immune surveillance and antigen recognition [79,80]. Continuous exposure to microbial communities within this niche may dynamically influence local immune responses, reinforcing the concept of acne as a disorder arising from complex host–microbe interactions.

In parallel, recent advances highlight the relevance of hair follicle stem cells (HFSCs) and sebaceous gland dynamics in linking inflammation and tissue repair. Upper HFSC populations and sebaceous gland progenitor cells contribute to sebocyte differentiation and wound healing processes [81], suggesting that acne-related inflammation may share biological pathways with regenerative responses. This integrated perspective—connecting microbiome composition, immune regulation, and follicular biology—provides a more comprehensive framework for understanding acne pathogenesis, as well as its variability in progression, resolution, and sequelae.

While several foundational aspects of microbiome involvement in acne are well established, many of the proposed therapeutic strategies remain in early stages of development. Interventions such as topical prebiotics, bacteriotherapy, phage therapy, and vaccine-based approaches are promising, yet require validation through robust, well-designed clinical trials.

Future research should therefore prioritize the generation of high-quality evidence to clarify the efficacy and safety of these strategies, enabling their appropriate integration into clinical practice. Such advances have the potential to meaningfully refine acne management while preserving the integrity and functional balance of the cutaneous ecosystem.

Despite promising advances, several aspects of microbiome-targeted interventions remain insufficiently understood, including long-term effects, patient variability, and reproducibility across populations. Conflicting findings in the literature further highlight the need for cautious interpretation.

## Figures and Tables

**Table 1:** Statements selected after the literature review and discussion group.

Consensus Statements with ≥75% Agreement During the First E-Survey	
**Agreement (%)**	The microbiome is an important contributor to acne pathogenesis.
	Skincare can impact the microbiome in acne.
	The exposome (lifestyle, climate, diet, pollution) has a direct impact on the microbiome in acne.
**100**	Microbiome-targeted treatments should be adopted to manage acne.
**89**	The microbiome influences acne severity.
	The GUT–SKIN axis has a direct impact on the microbiome in acne.
	Probiotics can improve skin microbiome balance and acne treatment efficacy.
	The inclusion of prebiotics, probiotics, and post-biotics can benefit acne therapy.
**78**	Microbiome-targeted strategies should be adopted as primary prevention of acne.
**75**	Microbiome-targeted strategies should be adopted as maintenance to prevent acne relapses.
**Consensus statements with ≥75% agreement during the second e-survey**	
**100**	The microbiome influences acne-induced hyperpigmentation.
	Topical prebiotic inclusion in acne treatment has a positive influence in the treatment.
**89**	The microbiome influences acne-induced scarring.
	Dietary habits, particularly high-glycaemic diets, can influence the composition of the skin microbiome and acne pathogenesis.

## Data Availability

No new data were created or analyzed in this study. Data sharing is not applicable to this article.
